# Polymorphism analysis in estrogen receptors alpha and beta genes and their association with infertile population in Pakistan

**DOI:** 10.17179/excli2015-559

**Published:** 2015-10-08

**Authors:** Sinha Liaqat, Shahida Hasnain, Saima Muzammil, Sumreen Hayat

**Affiliations:** 1 Department of Microbiology, Government College University, Faisalabad, Pakistan; 2 Department of Microbiology and Molecular Genetics, University of the Punjab, Lahore, Pakistan; 3 The Women University, 60000, Multan, Pakistan

**Keywords:** estrogen receptor, infertility, polymorphism, haplotype, genetic, linkage disequilibrium

## Abstract

Studies on polymorphism of estrogen receptor (ESR) alpha and beta genes have been mostly implicated in infertility, but the results have been controversial due to lack of comprehensive data. The present study focused on association of ESR genes with both male and female infertility. In *ESR*α, *Pvu*II (rs2234693) and *Xba*I (rs9340799) were studied while in *ESR*β gene, risk of infertility was determined for silent G/A *RsaI* (rs1256049) polymorphism. Total 124 subjects (74 cases and 50 controls) were part of this study having primary infertility. Restriction fragment length polymorphism (RFLP) was performed with *Pvu*II, *Xba*I and *Rsa*I to determine polymorphism. Correlation between age and follicle stimulating hormone (FSH) of cases and controls was determined and no association was found between infertility and FSH hormone. Heterozygous AG genotype of *Xba*I polymorphism (*P*= 2.505e-06) and heterozygous TC genotype (*P=* 0.00003) in *Pvu*II polymorphism were strongly associated with risk of infertility. In *ESR*β gene, there was lack of polymorphism for *Rsa*I in our population as all subjects were homozygous (GG). Haplotype frequencies showed that *Xba*I and *Pvu*II polymorphisms are in strong linkage disequilibrium. This study shows that in our population *Xba*I and *Pvu*II polymorphisms of *ESR*α are associated with risk of infertility.

## Introduction

Infertility has become a common problem as it is affecting almost 1 in 6 couples. It is defined as the incapability to achieve fertilization after a certain period of time following sexual intercourse (mostly 1 year or 6 months in certain cases) without any contraceptive measures (Brugo-Olmedo et al., 2001[4]). Infertility cannot be associated with a specific gender as both male and female factors account for 40 % while 20 % cases are either due to shared or unexplained causes (Bhatti et al., 1999[3]). In Pakistan, overall prevalence of infertility is about 22 %, out of which 5 % cases are considered as primary infertility whereas 18 % as secondary infertility (Herz, 1989[9]).

Numerous genes are involved in the complex process of reproduction. A qualitative diagnosis of infertility could only be done by focusing on female and male physical abnormalities, endocrine irregularities and genetic conditions of both partners associated with reproduction (Mak and Jarvi, 1996[17]).

One of the most common factors is mutation of genes expressed in hypothalamus and leads to low production of serum gonadotrophin such as follicle stimulating hormone (FSH) and luteinizing hormone (LH) which consequently leads to deficient or absence of puberty (Layman, 2002[14]). Estrogen action is observed both on peripheral and central nervous system which is mediated by estrogen receptors (ER). Estrogen receptors α (*ESR*α, *ESR*1) and β (*ESR*β, *ESR*2) genes are involved in controlling the physiological response to estrogen. *ESR*α gene is present on chromosome 6q25. It consists of 8 exons which are parted by 7 intronic regions having a complete size of 140 kb (Ponglikitmongkol et al., 1988[24]). The *ESR*β gene is located on chromosome 14q22-24, comprising 8 exons and covers approximately 40 kb (Enmark et al., 1997[8]). ESRα codes for a 595 amino acid protein (Menasce et al., 1993[18]), while ESRβ codes for a 530 amino acid protein (Ogawa et al., 1997[22]).

Most widely studied polymorphisms of ESRα involve *Pvu*II (rs2234693) and *Xba*I (rs9340799). These are found in the intron 1 and divided by only 46 bp. The *Pvu*II (T397C) polymorphism occurs due to T/C transition in first intron, while the *Xba*I (G351A) polymorphism is caused by a G/A transition located 50 base pairs downstream of the *Pvu*II polymorphic site (Keene et al., 2008[11]; Pollak et al., 2004[23]; Shearman et al., 2004[29]). Several sequence variants of the *ESR*β gene have also been identified which include 2 silent G/A polymorphisms, *Rsa*I (rs1256049) and *Alu*I (rs4986938) (Rosenkranz et al., 1998[25]). The expression of both these receptors has been recorded in testis and epididymis (O'Donnell et al., 2001[20]).

In human testis, ERα and ERβ have also been found in ejaculated spermatozoa (Durkee et al., 1998[6]; Lambard et al., 2004[13]). Absence of ER-α has been shown to lead to reduced epididymal sperm content. It also affects motility and fertilizing capacity (Eddy et al., 1996[7]; Ogawa et al., 1997[22]). In males *ESR*1 *Xba*I polymorphism was suggested to have an effect on azoospermia or idiopathic severe oligospermia while in women* ESR*1* Pvu*II strongly affected pregnancy rate after *in vitro* fertilization (IVF) (Kukuvitis et al., 2002[12]). *Pvu*II polymorphism is also reported to be associated with predisposition to endometriosis (Hsieh et al., 2007[10]) and controlled ovarian hyperstimulation (COH)/ pregnancy outcome of IVF. 

Association of *Rsa*I polymorphism (rs1256049) with male infertility has been established in Caucasian patients. It is located in exon 5 of ERβ (Aschim et al., 2005[1]). Several studies have been performed to estimate the distribution of various *ESR*1 gene polymorphisms and their association with female and male infertility. 

In a review article by O'Flynn O'Brien et al. (2010[21]) on the genetic causes of male factor infertility they concluded that there is a need for further examination of ER genes polymorphisms to reproduce the results of previous uncoordinated studies and to better explain the influence of these polymorphisms on male fertility (O'Flynn O'Brien et al., 2010[21]; Safarinejad et al., 2010[26]). 

The aim of this study is to determine the importance of *ESR*α and *ESR*β polymorphisms in the etiology of male and female infertility. To the best of our knowledge, there is no study so far in international literature, associating *ESR*α and *ESR*β polymorphisms with both male and female infertility.

## Materials and Methods

### Primary selection of subjects

For polymorphic analysis of ESRα and β gene, total of 124 individuals (both male and female) were included in this study. Blood samples of 74 infertile patients were collected from LIFE (Lahore Institute of Infertility and Endocrinology, Lahore-Pakistan). Every participant has completed detailed questionnaire, regarding their medical and surgical history, lifestyle habits, exposure to gonadotrophins and family history. The control group consisted of 50 subjects (both males and females). Males had normal semen parameters i.e. sperm count over 15 million sperm/ml according to WHO guideline (WHO, 1991[32]) of which had fathered at least one child without assisted reproductive technologies.

### Sampling

Each subject donated 5 ml of blood for genomic DNA extraction and serum analysis. 2 ml of each peripheral blood sample was transferred to a sterile EDTA coated vacuotainer (BD Vacutainer®, Catalog number: 367841) and remaining 3 ml was transferred into sterile tubes without having anti-coagulant for separation of serum (BD Vacutainer®, Catalog no. 368774). Serum was separated immediately after sample collection and stored at -20 °C until hormone profiling whereas blood vials were properly labeled and stored at -20 °C. 

An inclusion and exclusion criteria was set to carefully evaluate the patients in order to identify the exact cause and to select only primary infertile people. All male patients underwent semen analysis according to WHO's recommendations (WHO, 1991[32]) in respective hospital's lab and were categorized as azospermia, asthenospermia, oligospermia and oligonecrospermia. In female patients, presence of ovulation was confirmed by measurements of serum progesterone in mild luteal phase. Tubal patency was also investigated by hysterosalpinography. Endocrine hormone, i.e. FSH level of patients were also measured. General exclusion criteria included chemotherapy, radiotherapy and illness. Individuals with history of karyotypes abnormality, cystic fibrosis, AIDS, hepatitis or any other contagious/viral infections were excluded. The women giving history of corrective surgery of vagina and uterus were excluded.

Serum FSH was measured by a microplate immune enzymatic assay using immunoassay kit, catalog number: BC-1029 (BioCheck, Inc. Foster City, Canada). Standard protocol as mentioned in the kit was used for all samples.

### Extraction of genomic DNA 

For isolation of genomic DNA from whole blood Wizard^®^ Genomic DNA Purification Kit, (Promega) was used. Manufacturer recommendations were followed for isolation of DNA.

### ER-α genotype determination

For determination of ERα genotype, polymerase chain reaction restriction fragment length polymorphism (PCR-RFLP) analysis was performed. For determination of *Pvu*II (c.454 -397 T > C) and *Xba*I (c.454 -351 A>G) polymorphisms, PCR amplification of the polymorphic regions was performed using oligonucleotide primers as described elsewhere (Safarinejad et al., 2010[26]). For PCR amplification, total volume of reaction mixture was 50 µl which consisted of 25 µl master mix (Fermentas #K0171), 1 µl (10 pmol) of each forward and reverse primer and 5 µl of template DNA. To differentiate c.454-397 T/C (*Pvu*II) and c.454-351 A/G (*Xba*I) polymorphisms, digestion of the amplified PCR fragment of 1372 bp was performed with *Pvu*II (Fermentas#ER0631) and *Xba*I (Fermentas#ER0681) restriction enzymes, separately. The reaction mixture was mixed gently and spins down for few seconds. Then the reaction vials were kept at 37 °C water bath for 1 hour and stored at -20 °C until visualization on ethidium bromide stained 2.0 % agarose gel by gel doc.

### ERβ genotype determination

The *Rsa*I (1082 G>A) polymorphism in ERβ gene was identified by PCR amplification and restriction enzyme digestion. Total volume of reaction mixture for PCR amplification was 25 µl consisting of 12.5 µl of master mix (fermentas #K0171), 2 µl (32 pmol) of each forward and reverse primer, and 2 µl of template DNA. For restriction digestion, the amplified fragment was digested with *Rsa*I (Fermentas#ER1121). The reaction mixture was mixed gently and spins down for few seconds. Then the reaction vials were kept at 37°C water bath for 1 hour and stored at -20°C until visualization on ethidium bromide stained 2.0 % agarose gel. The primers, PCR conditions, restriction enzymes, and DNA fragment sizes are listed in Table 1(Tab. 1).

### Statistical analysis

The significance of differences in the genotype distribution and allele frequency were tested using the χ2 test, which was also used to determine the degree of subjects conforming to Hardy-Weinberg equilibrium, single genotype and the frequency of allele between each group. Clinical variables were compared using the independent sample t-test. An Odds Ratio (OR) at 95 % confidence intervals (CI) was calculated with respect to the presence of the reference genotype. The association between the combined genotypes of the *ER*α and *ER*β genes polymorphisms and the risk of infertility was also evaluated using Odds Ratio at 95 % confidence intervel. Haplotype construction and haplotype frequencies were determined by using the Haploview software version 3.2 available at http://www.hapmap.org. Linkage disequilibrium analysis was also performed with the Haploview program. The level of significance was set at P < 0.05. Data were analyzed with SPSS 16.0 for Windows (SPSS, Chicago, USA) software.

## Results

### Patient characteristics

Classifications of patients on basis of types of infertility are described in Table 2(Tab. 2). Briefly, in infertile males, azospermia was the most frequent cause of infertility (0.43). In infertile females the major cause of infertility was tubal factor (0.24) and sec tubal factor (0.23).

**Figure 1:**
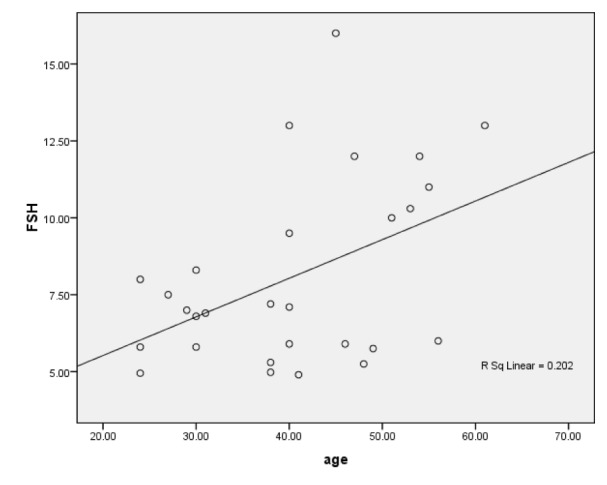
Scatter plot of positive linear correlation between age and FSH of fertile female subjects

No correlation was found between age of male and female patients and their endocrine levels. However a positive collinear relationship was found between the age and FSH of female controls where r = 0.450 (*P=*0.016) (Figure 1(Fig. 1)). In all cases significance level was at P ≤ 0.05.

### ESRα genotypes

Genotype and allele frequencies in *ESR α* variant polymorphism and their association with Hardy-Weinberg equilibrium (HWE) are given in Table 3(Tab. 3). For *Pvu*II polymorphism, there was significant deviance from HWE for cases (χ^2^=21.82, *P*< 0.001) but no observable deviance from HWE for controls (χ^2^=6.33, *P*=0.05) was determined. For *Xba*I polymorphism, there was evident deviance from HWE for both cases (χ^2^=26.19, *P*< 0.001) and controls (χ^2^=9.64, *P*=0.01) (Table 3(Tab. 3)).

Allelic frequency for all groups was also calculated and it further conformed to Hardy-Weinberg equilibrium. The tests for association adapted from Sasieni (1997[28]) were performed to find the association of allele frequency or genotype frequency of *Xba*I and *Pvu*II polymorphism with the risk of infertility. Odds Ratio (OR), 95 % Confidence interval (C.I.) and χ2 values were determined (Table 4(Tab. 4)). The significance level was set at P<0.05. In case of *Xba*I polymorphism, no significant association was found between allele A (*p=*0.83783) and G (*p =*0.83783) of cases and controls with risk of infertility. However heterozygous AG genotype showed strong association with risk of infertility in cases when compared to homozygous AA (*p*=2.505e-06) as well as rare homozygous GG genotype of controls. Rare homozygous GG (*p=*0.97111) and homozygous AA (*p=*0.97111) genotype of cases showed no significant association with risk for infertility. Individuals with genotype of AG and of GG (*p=*0.00069) in cases showed significant association with risk of infertility. This showed strong association of heterozygosity with risk of infertility in our population (Table 4(Tab. 4)). 

**Figure 2:**
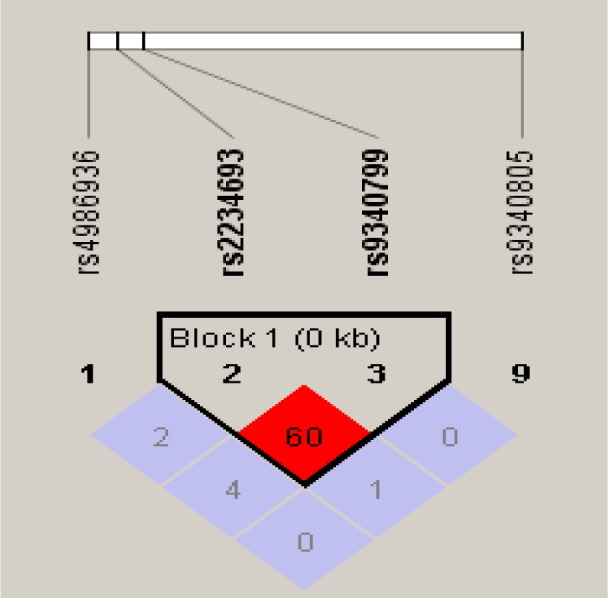
Linkage disequilibrium comparison of *Pvu*II (rs2234693) and *Xba*I (rs9340799) in *ESR*α gene

In case of *Pvu*II polymorphism, although there was difference between T and C allele frequencies in cases and controls (Table 4(Tab. 4)), but this variance was not statistically significant (*p=*0.76013). The heterozygous TC genotype of *Pvu*II showed more prevalence in infertile group and was significantly associated with risk of infertility when compared with homozygous TT (*p=*0.00003) and rare homozygous CC (*p=*0.00005) genotype of cases and controls. Homozygous TT (*p=*0.84154) and rare homozygous CC genotype of cases showed no significant association with risk of infertility. Allele positivity was determined by determining the number of individuals possessing a particular genotype in cases and control group. Infertile subjects with heterozygous genotype TC when combined with infertile subjects having rare homozygous genotype CC (*p=*0.00158), showed significant association with risk of infertility (Table 4(Tab. 4)). Haplotype frequencies and analysis of linkage disequilibrium was performed by using haploview program. Strong linkage disequilibrium was found between the *Pvu*II and *Xba*I polymorphisms of the *ESR*α gene (D'=1.0, LOD=0.37, r-squared=0.013) (Figure 2(Fig. 2)).

### ESRβ genotype

*Rsa*I digestion yielded one band of 156 bp in the normal ERβ sequence (GG). All the cases and controls were analyzed for this polymorphism, but no polymorphism was detected in any subject (both patients and controls). All the subjects were homozygous for common allele GG. This indicates absence of this polymorphism in our studied population.

## Discussion

In the present study 74 patients (cases) with primary infertility were selected while 50 healthy fertile individuals were considered as controls. In infertile male cases, azospermia was the most frequent cause of infertility (43 %) while in infertile females the major cause of infertility was tubal factor (24 %) and sec tubal factor (23 %). These results correspond to former epidemiological studies which have shown that in a sub fertile population male factor infertility has been observed in 25 %-35 % couples. About 14-22 % have tubal factor, 10-27 % have ovulatory dysfunction, 5-6 % have endometriosis while 10-17 % have unexplained causes of infertility (Maheshwari et al., 2008[16]). 

Correlation between age and FSH of patients and controls was calculated. No correlation was found between age of male and female patients and their endocrine levels. In our work lack of correlation between age and serum FSH levels of cases reveals that the study subjects did not had gonadotrophins related infertility. However a positive collinear relationship was found between the age and FSH of female controls. The increase in basal FSH can be considered as the earliest endocrine marker of reproductive aging (Burger et al., 2007[5]). 

In this case-control study the association between *Xba*I and *Pvu*II polymorphism of the *ESRα* gene and *Rsa*I polymorphism of *ESR*β gene with fertility status was analyzed. Previous studies on the influence of the ERα and ERβ genes involved in male factor infertility have yielded conflicting results (Tüttelmann et al., 2007[31]; O'Flynn O'Brien et al., 2010[21]; Meng et al., 2013[19]). Assessment of deviance from HWE in case of disease association is significantly relevant for many studied genes especially if the functional variants can be directly genotyped. For *Pvu*II polymorphism, there was significant deviance from HWE for cases (χ^2^=21.82, *P*<0.001) but there was no observable deviance from Hardy-Weinberg equilibrium for controls (χ^2^=6.33, *P*=0.05). Salanti et al. (2005[27]) had suggested in their studies that in occurrence of an association, cases need not be in HWE. They proposed that screening of effected individuals with HWE is a comparatively effective method for detection of gene-disease associations. If a locus is not deviating from HWE then various conditions are being fulfilled. These include lack of recent mutations and genetic drift as well as adaption to Mendelian segregation and random mating. According to Li and Li (2008[15]), for case-control studies as the prevalence of disease in controls is markedly less so they are considered more alike to healthy population and thus give evidence about departure towards HWE. But when cases are more different from general population, they contribute information about genotype relative risk estimation.

In present investigation for *Xba*I polymorphism, there was observable deviance from Hardy-Weinberg equilibrium for both cases (χ^2^=26.19, *P*<0.001) and controls (χ^2^=9.64, *P*=0.01). Allelic frequency for all groups was also calculated and it conformed to Hardy-Weinberg equilibrium. Previous investigations have revealed that in approximately 10 % of gene-disease association studies, there is significant deviation from HWE in genotype frequencies of healthy controls. Only 5 % of these are expected by chance alone even at significance level of P=0.05 with sufficiently powered studies as most of these studies use small underpowered sample sizes (Trikalinos et al., 2006[30]).

In case of *Xba*I polymorphism, insignificant association was found between allele A (*p=*0.83783) and G (*p=*0.83783) of cases and controls with risk of infertility. However heterozygous genotype AG (*p*=2.505e-06) showed strong association with risk of infertility in cases when compared to homozygous AA as well as rare homozygous GG genotype of controls. Rare homozygous GG and homozygous AA (*p=*0.97111) genotype of cases showed no significant association with risk for infertility.

These results are in contradiction to the study by Ayvaz et al. (2009[2]) in Turkish population which described a significant correlation in *Xba*I polymorphism and chances of infertility. However in their population the GG genotype (OR 6.75, 95 % CI: 2.11- 21.59) showed about sevenfold increased predisposition to infertility compared to individuals carrying homozygote AA, whereas insignificant disposition was found in heterozygous AG (OR 1.53, 95 % CI: 0.86-2.73) individuals. They also detected a significant difference in allele frequencies of *Xba*I polymorphism in cases and controls (Ayvaz et al., 2009[2]). In the study by Safarinejad et al. (2010[26]) on infertile men in Iranian population, allele frequency of A was lower in controls than in infertile men and this difference was statistically significant (*p*=0.048). However in their population the homozygous AA genotype of *Xba*I polymorphism was associated with risk of infertility due to its prevalence in infertile group as compared to controls (*p*=0.014). Allele positivity was determined by determining the number of individuals possessing a particular genotype in affected and control group. Individuals with genotype of AG and of GG (OR=4.931, C.I.=1.862-13.058, *p=*0.00069) in cases showed significant association with risk of infertility. This showed strong association of heterozygosity with risk of infertility in our population for *Xba*I polymorphism. 

In case of *Pvu*II polymorphism, although there was difference between T and C allele frequencies in cases and controls, but this difference was statistically insignificant (*p =*0.76013). The heterozygous TC genotype of *Pvu*II was more common in infertile group and was significantly associated with risk of infertility when compared with homozygous TT (*p=*0.00003) and rare homozygous CC (*p=*0.00005) genotype of cases and controls. Homozygous TT (*p =*0.84154) and rare homozygous CC genotype of cases showed no significant association with risk of infertility. Individuals for determination of allele positivity with heterozygous genotype TC when combined with cases having rare homozygous genotype CC (*p=*0.00158), showed significant association with risk of infertility. Thus in our population individuals with heterozygous TC genotype are more predisposed to infertility as compared to individuals with CC or TT genotype. These results varied from those reported in other populations. In Turkish population, for *Pvu*II polymorphism, a detectable association was found between fertile and infertile group for heterozygous TC and rare homozygous CC genotype (Ayvaz et al., 2009[2]). In the study on infertile men in Iranian population, subjects having the *Pvu*II CC genotype had reduced risk of infertility as compared to subjects having the *Pvu*II TT genotype (OR = 0.56, 95 % CI = 0.26-0.80; P = 0.011) (Safarinejad et al., 2010[26]). 

Strong linkage disequilibrium existed between polymorphisms of *Xba*I and* Pvu*II of *ESR*α gene (D'=1.0). Thus the association observed in these polymorphisms and infertility could be due to only one of them. As a multifactorial condition like infertility is dependent on several polymorphisms, so a third polymorphism in strong linkage disequilibrium with *Xba*I and *Pvu*II can be the actual cause of this association. The genomic organization of *ESR*α gene promoter is very complex. Alternative splice sites are present in multiple promoter regions which results in formation of different estrogen protein transcripts. This difference in expression patterns can affect the function and production of *ESR* protein. Hence the possibility of these polymorphisms affecting the correct splicing of RNA and producing alternatively spliced mRNA variants cannot be ruled out (Ayvaz et al., 2009[2]; Safarinejad et al., 2010[26]) 

For *Rsa*I polymorphism all the cases and controls were analyzed, but no polymorphism was detected in any subject (both patients and controls). All the subjects were homozygous for common allele GG. This indicates absence of this polymorphism in our studied population.

In conclusion this work showed significant association between *Xba*I and *Pvu*II polymorphisms with risk of infertility. Also detection of significant deviance from Hardy-Weinberg raised several possibilities about this study and the population from which it is derived. Pursuing this course can lead to more useful information regarding ESR gene. More thorough examination of ESR locus is necessary to determine whether other regions of *ESR*α and *ESR*β may also have functional significance related to infertility.

## Acknowledgement

The authors acknowledge the Department of Microbiology and Molecular Genetics, University of the Punjab, Lahore, Pakistan, which provided the facilities to carry out this research. The authors would also like to thank the participants’ families for their support and Hameed Latif hospital for collection of blood samples.

## Potential conflict of interest

None declared.

## Figures and Tables

**Table 1:**
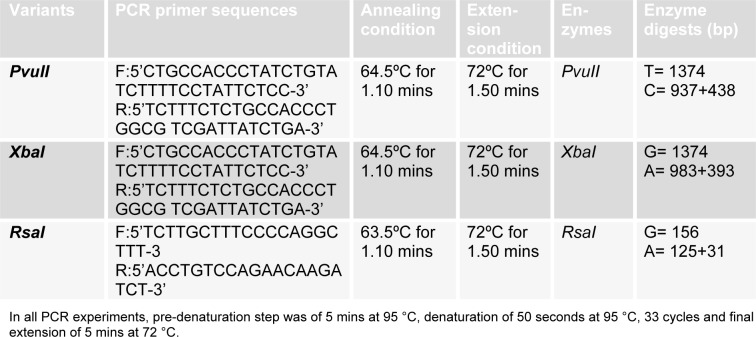
Primers, PCR conditions and restriction enzymes for estrogen receptor SNPs

**Table 2:**
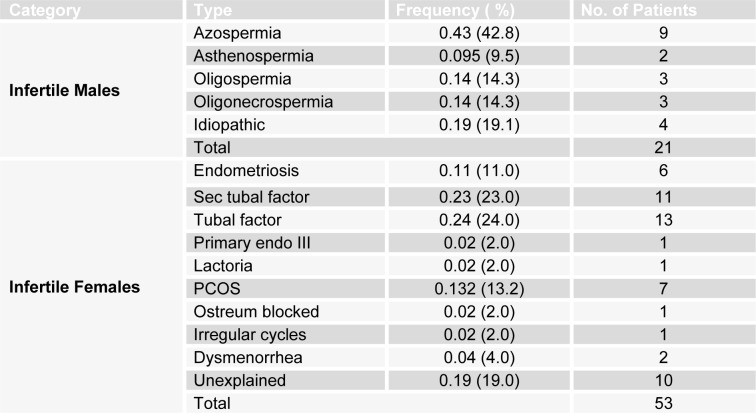
Classification of male and female patients on the basis of various type of infertility

**Table 3:**
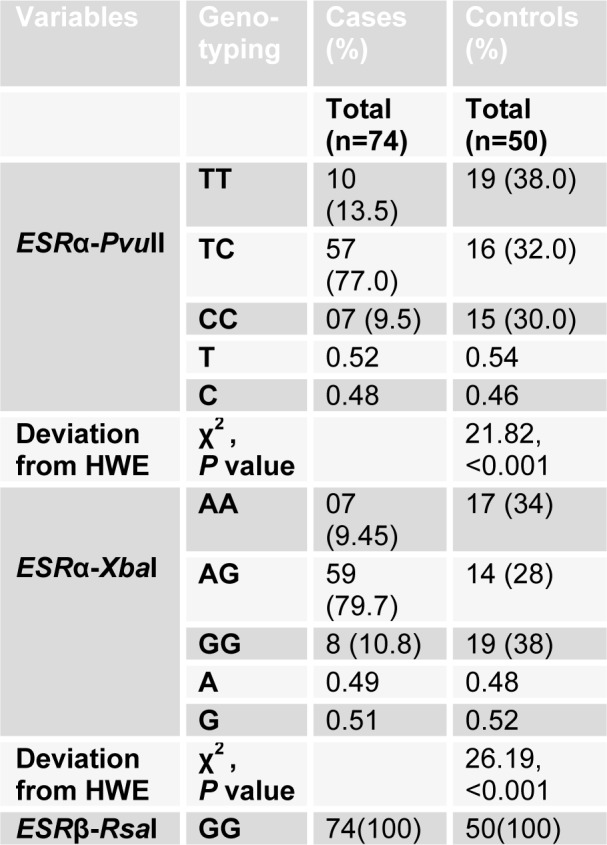
Frequency distribution of *ESR*α genotype, alleles and their association with Hardy-Weinberg equilibrium (HWE)

**Table 4:**
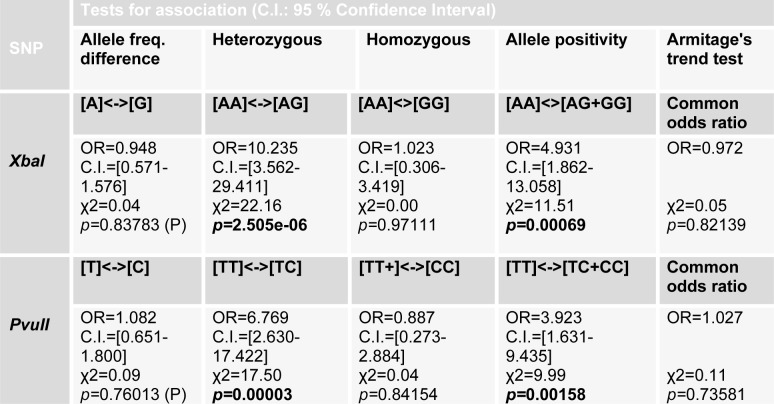
Tests for association of allele frequencies and genotypes of *ESR*α, *Xba*I and *Pvu*II with risk of infertility

## References

[1] Aschim EL, Giwercman A, Ståhl O, Eberhard J, Cwikiel M, Nordenskjöld A (2005). The RsaI polymorphism in the estrogen receptor-beta gene is associated with male infertility. J Clin Endocr Metab.

[2] Ayvaz OU, Ekmekçi A, Baltaci V, Onen HI, Unsal E (2009). Evaluation of in vitro fertilization parameters and estrogen receptor alpha gene polymorphisms for women with unexplained infertility. J Asist Reprod Gen.

[3] Bhatti LI, Fikree FF, Khan A (1999). The quest of infertile women in squatter settlements of Karachi, Pakistan: a qualitative study. Soc Sci Med.

[4] Brugo-Olmedo S, Chillik C, Kopelman S (2001). Definition and causes of infertility. Reprod BioMed Online.

[5] Burger HG, Hale GE, Robertson DM, Dennerstein L (2007). A review of hormonal changes during the menopausal transition: focus on findings from the Melbourne Women's Midlife Health Project. Hum Reprod Update.

[6] Durkee TJ, Mueller M, Zinaman M (1998). Identification of estrogen receptor protein and messenger ribonucleic acid in human spermatozoa. Am J Obstet Gynecol.

[7] Eddy EM, Washburn TF, Bunch DO, Goulding EH, Gladen BC, Lubahn DB (1996). Targeted disruption of the estrogen receptor gene in male mice causes alteration of spermatogenesis and infertility. Endocrinology.

[8] Enmark E, Pelto-Huikko M, Grandien KAJ, Lagercrantz S, Lagercrantz J, Fried G (1997). Human estrogen receptor beta-gene structure, chromosomal localization, and expression pattern. J Clin Endocr Metab.

[9] Herz EK (1989). Infertility and bioethical issues of the new reproductive technologies. Pediatr Clin North Am.

[10] Hsieh YY, Wang YK, Chang CC, Lin CS (2007). Estrogen receptor α 351 XbaIG and 397 PvuIIC-related genotypes and alleles are associated with higher susceptibilities of endometriosis and leiomyoma. Mol Hum Reprod.

[11] Keene KL, Mychaleckyj JC, Smith SG, Leak TS, Perlegas PS, Langefeld CD (2008). Comprehensive evaluation of the estrogen receptor alpha gene reveals further evidence for association with type 2 diabetes enriched for nephropathy in an African American population. Hum Genet.

[12] Kukuvitis A, Georgiou I, Bouba I, Tsirka A, Giannouli CH, Yapijakis C (2002). Association of oestrogen receptor alpha polymorphisms and androgen receptor CAG trinucleotide repeats with male infertility: a study in 109 Greek infertile men. Int J Androl.

[13] Lambard S, Galeraud-Denis I, Saunders PT, Carreau S (2004). Human immature germ cells and ejaculated spermatozoa contain aromatase and oestrogen receptors. J Mol Endocrinol.

[14] Layman LC (2002). Human gene mutations causing infertility. J Med Genet.

[15] Li M, Li C (2008). Assessing departure from Hardy Weinberg equilibrium in the presence of disease association. Genet Epidemiol.

[16] Maheshwari A, Hamilton M, Bhattacharya S (2008). Effect of female age on the diagnostic categories of infertility. Hum Reprod.

[17] Mak V, Jarvi KA (1996). The genetics of male infertility. J Urol.

[18] Menasce LP, White GRM, Harrison CJ, Boyle JM (1993). Localization of the estrogen receptor locus (ESR) to chromosome 6q25. 1 by FISH and a simple post-FISH banding technique. Genomics.

[19] Meng J, Mu X, Wang YM (2013). Influence of the XbaI polymorphism in the estrogen receptor-α gene on human spermatogenic defects. Genet Mol Res.

[20] O'Donnell L, Robertson KM, Jones ME, Simpson ER (2001). Estrogen and spermatogenesis. Endocr Rev.

[21] O'Flynn O'Brien KL, Varghese AC, Agarwal A (2010). The genetic causes of male factor infertility: a review. Fertil Steril.

[22] Ogawa S, Lubahn DB, Korach KS, Pfaff DW (1997). Behavioral effects of estrogen receptor gene disruption in male mice. Proc Natl Acad Sci USA.

[23] Pollak A, Rokach A, Blumenfeld A, Rosen LJ, Resnik L, Pollak RD (2004). Association of oestrogen receptor alpha gene polymorphism with the angiographic extent of coronary artery disease. Eur Heart J.

[24] Ponglikitmongkol M, Green S, Chambon P (1988). Genomic organization of the human oestrogen receptor gene. EMBO J.

[25] Rosenkranz K, Hinney A, Ziegler A, Hermann H, Fichter M, Mayer H (1998). Systematic mutation screening of the estrogen receptor beta gene in probands of different weight extremes: identification of several genetic variants. J Clin Endocrinol Metab.

[26] Safarinejad MR, Shafiei N, Safarinejad S (2010). Association of polymorphisms in the estrogen receptors alpha, and beta (ESR1, ESR2) with the occurrence of male infertility and semen parameters. J Steroid Biochem.

[27] Salanti G, Amountza G, Ntzani EE, Ioannidis JPA (2005). Hardy Weinberg equilibrium in genetic association studies: an empirical evaluation of reporting, deviations, and power. Eur J Hum Genet.

[28] Sasieni PD (1997). From genotypes to genes: doubling the sample size. Biometrics.

[29] Shearman AM, Cupples LA, Demissie S, Peter I, Schmid CH, Karas RH (2004). Association between estrogen receptor [alpha] gene variation and cardiovascular disease. Obstet Gynecol Surv.

[30] Trikalinos TA, Salanti G, Khoury MJ, Ioannidis JPA (2006). Impact of violations and deviations in Hardy-Weinberg equilibrium on postulated gene-disease associations. Am J Epidemiol.

[31] Tüttelmann F, Rajpert-De Meyts R, Nieschlag E, Simoni M (2007). Gene polymorphisms and male infertility – a meta-analysis and literature review. Reprod Biomed Online.

[32] WHO, World Health Organization (1991). Infertility: a tabulation of available data on prevalence of primary and secondary infertility. Programme of WHO for Maternal-Child Health, Family Planning.

